# 20*S*-Hydroxyvitamin D3, a Secosteroid Produced in Humans, Is Anti-Inflammatory and Inhibits Murine Autoimmune Arthritis

**DOI:** 10.3389/fimmu.2021.678487

**Published:** 2021-06-30

**Authors:** Arnold E. Postlethwaite, Robert C. Tuckey, Tae-Kang Kim, Wei Li, Syamal K. Bhattacharya, Linda K. Myers, David D. Brand, Andrzej T. Slominski

**Affiliations:** ^1^ Research Service, Department of Veterans Affairs Medical Center, Memphis, TN, United States; ^2^ Department of Medicine, University of Tennessee Health Science Center, Memphis, TN, United States; ^3^ School of Molecular Sciences, The University of Western Australia, Perth, WA, Australia; ^4^ Department of Pathology, University of Alabama at Birmingham, Birmingham, AL, United States; ^5^ Research Service, Department of Veterans Affairs Medical Center, Birmingham, AL, United States

**Keywords:** vitamin D, arthritis, RA, 20S(OH)D_3_, mouse, collagen

## Abstract

The ability to use large doses of vitamin D3 (D3) to chronically treat autoimmune diseases such as rheumatoid arthritis (RA) is prohibitive due to its calcemic effect which can damage vital organs. Cytochrome P450scc (CYP11A1) is able to convert D3 into the noncalcemic analog 20*S*-hydroxyvitamin D3 [20*S*(OH)D3]. We demonstrate that 20*S*(OH)D3 markedly suppresses clinical signs of arthritis and joint damage in a mouse model of RA. Furthermore, treatment with 20*S*(OH)D3 reduces lymphocyte subsets such as CD4^+^ T cells and CD19^+^ B cells leading to a significant reduction in inflammatory cytokines. The ratio of T reg cells (CD4+CD25+Foxp3+ T cells) to CD3+CD4+ T cells is increased while there is a decrease in critical complement-fixing anti-CII antibodies. Since pro-inflammatory cytokines and antibodies against type II collagen ordinarily lead to destruction of cartilage and bone, their decline explains why arthritis is attenuated by 20(OH) D3. These results provide a basis for further consideration of 20*S*(OH)D3 as a potential treatment for RA and other autoimmune disorders.

## Introduction

Rheumatoid arthritis (RA) is one of the more common autoimmune diseases (affecting approximately 0.5-1% of the world’s population (1, 2). Although, multiple diathroidial joints are the main targets of autoimmune attack, other organ systems are also involved, reducing life expectancy by about 10 years in patients with RA (3, 4). Standard disease modifying anti-rheumatic drugs, (DMARDs), such as methotrexate (MTX) and “biologics” that target specific cytokines (e.g., TNFα) or surface molecules on immune cells (e.g., CTLA-4 on T cells), do reduce both joint damage and certain systemic complications of RA. However, their use occasionally triggers adverse events such as increased infections and development of certain neoplasms, other autoimmune diseases, or interstitial lung disease (5–8). Clearly, safer and effective therapeutic agents for RA are needed.

In humans, vitamin D3 (D3 aka cholecalciferol) is synthesized in the skin *via* ultraviolet B (UVB) induced photoconversion of 7-dehydrocholestrol (7-DHC), a precursor to cholesterol (9, 10). D3, which is inert as a pro-hormone, undergoes two hydroxylation steps: first in the liver (or skin itself) by a 25-hydroxylase (CYP2R1 or CYP27A1) to form 25(OH)D3, and then in the kidney and other tissues by 25-hydroxyvitamin D-1 alpha hydroxylase (CYP27B1) to form 1,25-dihydroxyvitamin D3 (1,25(OH)_2_D3) (11–15). 1,25(OH)_2_D3 exerts positive or negative influences on D3-dependent gene expression by binding to the ligand binding domain of the vitamin D receptor (VDR) (16). A heterodimer is then formed within the retinoic acid X receptor (RXR), which, (with the contributions of basal transcriptional machinery plus coactivators and corepressors) forms transcription complexes at vitamin D response elements (VDRE) of target genes (17).

1,25(OH)_2_D3 is the most extensively characterized active naturally occurring D3 metabolite that, not only systematically regulates calcium homeostasis and bone metabolism, but also possesses immunomodulatory properties. Clinically, normal D3 level is associated with better outcomes in patients with a variety of autoimmune diseases (18–21). In RA, disease activity, C reactive protein and disability scores are inversely related to serum levels of 25(OH)D, and anticyclic citrullinated peptide antibody positivity in RA patients is correlated with D3 insufficiency [25(OH)D, 21-29 ng/ml] and deficiency [25(OH)D <20 ng/ml] (18, 22–25). Furthermore, the VDR Fok1 polymorphism may confer susceptibility to RA in Europeans and Native Americans (26, 27). These observations suggest D3 may have salutary effects in RA. Earlier studies demonstrated 1,25(OH)_2_D3 inhibited arthritis in the type II collagen (CII)-induced arthritis (CIA) model of RA in mice fed a low calcium diet to protect against development of hypercalcemia (28). Unfortunately, 1,25(OH)_2_D3 or its precursors, 25(OH)D3 or D3 (cholecalciferol), induce hypercalcemic toxicity when given chronically at the pharmacological doses needed to maximally suppress arthritis and autoimmunity, limiting the amounts that can be given chronically to patients to treat autoimmune diseases such as RA.

We have discovered a novel pathway of D3 metabolism operative in humans, mediated by cytochrome P450scc (CYP11A1), which is modified by CYP27B1, that generates additional biologically active products (29–31). These are at least as potent as classical 1,25(OH)_2_D3 when tested *in vitro* and *in vivo* in several model systems and, like 1,25(OH)_2_D3, bind to the VDR (32–37). The main and first product of the pathway, 20*S*(OH)D3, in contrast to 25(OH)D3 or 1,25(OH)_2_D3, is nontoxic *(*i.e., noncalcemic and nontoxic to the hematopoietic system, liver, kidney, and heart) at doses as high as 60 µg/kg, while 25(OH)D3 or 1,25(OH)_2_D3 induce hypercalcemia at doses ≤2 µg/kg (38–41). Finally, 20S(OH)D3 is produced *in vivo*, being present in human serum at 1/20^th^ the concentration of classical 25(OH)D3 (31) and has also been detected in honey (42). The cytochrome SCC enzymes are unusual in that they hydroxylate vitamin D3 to produce 20S-hydroxyvitamin D3 [20(OH)D3] rather than cleaving the side chains of D3 (43). These properties define 20*S*(OH)D3 as a hither to unrecognized novel endogenous regulator/natural product.

In the present study, CIA in DBA/1 mice (the most widely studied animal model of RA) was used to collect preclinical data on 20*S*(OH)D3 as a potential treatment for human RA. When immunized with bovine CII, DBA/1 mice rapidly develop an anti-CII Th1 and Th17 T cell (IFNγ, IL-2, GM-CSF, TNFα, and IL-17) and later a B cell response characterized by production of IgG1 and complement fixing IgG2a antibodies to CII that triggers inflammation (44–49). At about 3 weeks post immunization, arthritis (which histologically resembles RA) begins to develop in peripheral joints (44, 46, 50–52). As time passes arthritis develops in more joints and with greater degrees of inflammation and damage. We demonstrated that treatment of mice with CIA with 20*S*(OH)D3 reduces the severity of clinical arthritis accompanied by reduction in joint destruction, in serum anti-CII antibodies, in lymphoid organ CD4^+^ T and CD19^+^ B cells, and production by cultured draining lymph node cells of TH1, TH17, and inflammatory cytokines and chemokines.

## Materials and Methods

### Production and Purification of 20(OH)D3

20*S*(OH)D3 was generated by enzymatic hydroxylation of D3 catalyzed by CYP11A1 as previously described by our group (29, 53). Briefly, 10 mM D3 in 45% 2-hydroxpropyl-β-cyclodextrin was prepared. Buffer comprising 20 mM HEPES (pH 7.4), 100 mM NaCl, 0.1mM dithiothreitol, 2 µM CYP11A1, 0.1 mM EDTA, 0.3 µM adrenodoxin reductase, 10 µM adrendoxin, 2 mM glucose 6-phosphate, 2U/ml glucose 6-phosphate dehydrogenase, and 50 µM NADPH was mixed with the D3 stock solution rendering a final D3 concentration of 200 µM and 0.9% concentration of 2-hydroxypropyl-β-cyclodexrtin. Following 8 min pre-incubation, the reaction was initiated by adding NADPH, after which the samples were incubated for 3 h at 37°C with gentle shaking. Ice cold dichloromethane (20 ml) was then added to stop the reactions after which the reaction products were extracted with dichloromethane as previously described (53–55). The 20(OH)D3 product was purified by preparative thin-layer chromatography, followed by reverse phase high performance liquid chromatography, as previously described (55). Routinely 0.3 mg of purified 20*S*(OH)D3 was recovered from 12.5 ml of the starting incubation mixture. Aliquots of the purified 20*S*(OH)D3 were dried under nitrogen and stored at -80°C until used.

#### Mice

Female DBA/1 Lac J mice, age 6 weeks old (Jackson Laboratories, Bar Harbour, ME) were housed in a pathogen-free AAALAC-approved animal care facility at the University of Tennessee Health Science Center (UTHSC) and Department of Veterans Affairs Medical Center (VAMC), Memphis, TN. Mice were fed regular laboratory chow and water ad libitam, and housed under a 12 h light and 12 h darkness cycle. Animal protocols for the study were approved by the Institutional Animal Care and Use Committees at UTHSC and VAMC Memphis.

### Induction and Assessment of CIA

Mice were immunized with native bovine CII prepared (as previously described from fetal calf articular cartilage) (47). Groups of 12 mice (for assessment of arthritis development) or groups of 5 or 6 mice (for other studies) were administered different amounts of 20*S*(OH)D3 dissolved in sterile sesame oil (S.O) or propylene glycol [(PG) (Sigma-Aldrich, St. Louis, MO)]. The S.O. or PG, with-or-without 20*S*(OH)D3, was administered daily intraperitoneally (i.p.) or *via* daily oral gavage in volumes of 50 µl and 100 µl, respectively. For studies using PG to solubilize 20*S*(OH)D3, the PG, with-or-without 20*S*(OH)D3, was diluted 1:5 by volume with sterile normal saline, and 100 µl was administered daily by oral gavage. Rheumatrex tablets (DAVA Pharmaceuticals, Inc., Fort Lee, NJ) were used as a source of methotrexate sodium. Methotrexate 2.5 mg/kg was administered in 100 µl normal saline weekly by oral gavage. The tablets were crushed and solubilized in sterile normal saline.

Arthritis severity was assessed in each paw every other day by two observers (one of whom was blinded to treatment) using the following scale: 0=no swelling or redness, 1=slight swelling and redness, 2=moderate swelling or redness, 3=marked swelling and redness, and 4=marked swelling and redness with some deformity (47). For histological assessment of joint tissue, mice were euthanized, all paws were removed, decalcified, processed and scored histologically, as previously described (56). Evaluation of each joint was done in a blinded manner using 4 parameters (0–3 scale for each parameter): synovial inflammation and thickness, synovial leukocyte invasion into the joint, cartilage unevenness caused by inflammation related cartilage damage, and subchondral bone erosion. The total histologic score represented the sum of the 4 parameters. The maximal histologic score per mouse paw was 12 and 48 per mouse.

### Quantitation of Anti-CII Antibodies in Sera

Specific murine IgG1, IgG2a, and Ig2b anti-CII antibodies were quantitated in sera using a commercial ELISA, according to the manufacturer’s instructions (Chondrex, Redwood, WA).

### Flow Cytometric Assessment of Lymphoid Cells

Isolated cells from spleen or draining para aortic, popliteal or inguinal lymph nodes were labeled as follows: Alexa-700 labeled rat anti-mouse CD3, PE-Cy7-labeled rat anti-mouse CD25, Per CP-Cy5.5-labeled rat anti-mouse CD4, FoxP3 Tregs were detected by FoxP3 staining kit, and FITC-labeled rat anti-mouse CD-19 (BD Bioscience, San Diego, CA). Flow cytometry was performed on a SORP BD LSRII instrument and results analyzed by FlowJo.

### Cytokine Quantitation

Draining lymph node cells were isolated and cultured at 2x10^6^ cells/ml in RPMI 1640 medium containing 9% fetal calf serum, penicillin 100 u/ml, streptomycin 100 µg/ml, 1% glutamax, 1% pyruvate, and 1% 2-mercaptoethanol. Supernatants were collected after 48 h or 120 h of culture and analyzed by multiplex sandwich immunoassay (Bio-plex mouse cytokine/chemokine kits, Bio Rad) for levels of different cytokines using a Luminex instrument according to the manufacturer’s protocol.

### Quantitation of Total Serum Calcium

Levels of calcium in mice serum were quantitated by atomic absorption spectroscopy as previously described (57).

### Statistical Analyses

Differences between groups were analyzed using 2 way RM ANOVA when multiple comparisons were made, Student’s 2-sample *t*-test was used for single comparisons between groups with normally distributed data, or by Mann-Whitney rank sum test for data not normally distributed. The level of significance was set at *P < 0.05.*

## Results

### 20S(OH)D3 Treatment Suppresses Development of CIA and Associated Joint Damage

**Figure 1:**
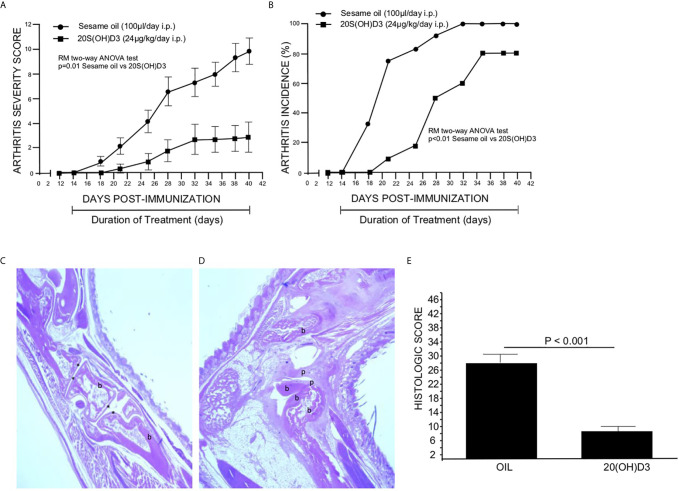
20*S*(OH)D3 suppresses CIA: 24 DBA/1 Lac J female mice at 6 wks of age were immunized with bovine CII and 14 days later 12 mice were treated daily with intraperitoneal (i.p.) 50 µl S.O. and 12 mice with 50 µl S.O. containing 2.4 µg/kg 20*S*(OH)D3. Arthritis severity **(A)** and incidence **(B)** were assessed by a blinded observer and each paw was scored on a scale of 0 to 4 with 16 being the maximal score per mouse. All mice survived to day 40. On Day 40, mice were euthanized, sera obtained, and paws processed for histology and scored histologically as described in Methods. An H&E stained section of the decalcified hind paw of a mouse treated with 20*S*(OH)D_3_ shows preservation of joint structures with minimal inflammation (10X magnification, b, bone, * identifies joint space) **(C)** while S.O.-treated mouse hind paws showed marked joint destruction with extensive loss of cartilage and bone with invasion of pannus (20X magnification, b, bone; p, pannus) **(D)**. 20*S*(OH)D3- treated CIA mice had a lower histologic score than S.O. treated CIA mice **(E)**. Photographs of typical paws for the scoring system we used are available online at the Hooke Laboratories Web Site (https://hookelabs.com/services/cro/cia/MouseCIAscoring.html).

To determine whether 20*S*(OH)D3 treatment started day 14 post CII immunization, after T cell priming to CII is firmly established, would suppress the development of CIA, two groups of mice (12 per group) were immunized with CII and from days 14-to-40 post immunization treated daily with i.p. injections of sterile 50 µl S.O. or 50 µl S.O. containing 20*S*(OH)D3 at a dose of 2.4 µg/kg/day. The 20*S*(OH)D3 treatment markedly reduced the mean arthritis severity score (**Figure 1A**) and arthritis incidence (**Figure 1B**). The curves comparing drug to vehicle are roughly parallel, suggesting that the greatest effect is on the magnitude of the immune response rather than elicitation of the response. The histologic scores reflecting inflammation, cartilage damage and subchondral bone erosion in joints harvested at sacrifice on Day 40 post CII immunization were significantly reduced in 20*S*(OH)D3 treated versus S.O. treated CIA mice (**Figure 1E**), reflecting the ability of 20(OH)D3 to protect joints from damage in this model.

Representative images of hematoxylin and eosin-stained sections of hind paws harvested on Day 40 post CII immunization show marked destruction of joint structures in S.O. treated CIA mice (**Figure 1D**), while joint structure was maintained in mice with CIA treated with *20S(OH)D3* (**Figure 1C**). Aliquots of sera were subjected to analysis of calcium content by atomic absorption spectroscopy. There was no difference in levels of serum calcium between 20*S*(OH)D3- and S.O.-treated mice with CIA (S.O. = 9.50 ± 0.50 mg/dL, 20S(OH)D3 = 9.57± 0.50 mg/dL, p = NS). This is compatible with our earlier report that 20*S*(OH)D3 does not induce hypercalcemia at doses up to 60 µg/kg when administered to C57BL/6 daily by i.p. injections for 21 days (39).

### 20S(OH)D3 Treatment Reduces Levels of Serum Antibodies to CII

**Figure 2:**
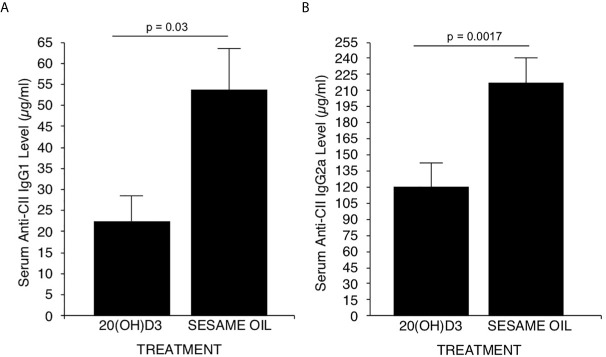
20(OH)D3 reduces serum levels of anti-CII antibodies in CIA mice: Aliquots of sera harvested at day 40 post-immunization with CII from mice described in **Figure 1A** were analyzed for levels of CII specific murine IgG1, IgG2a and IgG2b antibodies as described in Methods. 20*S*(OH)D3 treatment significantly reduced levels of anti CII IgG1 and IgG2a **(A, B)** but not of anti-CII IgG2b antibodies (data not shown).

Earlier studies demonstrated that the generation of complement fixing anti-CII antibodies is essential for development of CIA (58, 59). The major complement fixing anti-CII antibodies generated in DBA/1 mice immunized with CII are of the IgG2a subclass (59). However, the less potent complement fixing anti CII IgG1 and anti CII Ig G2b antibodies are also generated (59). Therefore, it was essential to assess whether 20*S*(OH)D3 versus S.O. vehicle treatment of mice immunized with CII developed less IgG2a and IgG1 anti-CII antibodies. On Day 40, sera from mice in **Figure 1A** were analyzed for anti-CII specific antibodies by a commercial ELISA kit from Chondrex specific for IgG1, IgG2a and Ig2b anti-CII antibodies. Significant reductions in serum levels of anti-CII antibodies of the IgG1 (**Figure 2A**) and IgG2a (**Figure 2B**) subclasses (*P*=0.017 and *P*=0.03), respectively occurred in 20*S*(OH)D3 treated mice compared to S.O. treated mice with CIA. However, IgG2b anti-CII antibody levels were not significantly different between 20*S*(OH)D3- and S.O.- treated cohort with CIA (data not shown). These reduced complement fixing anti-CII antibodies likely resulted in reduced arthritis severity observed in mice treated with CIA treated with 20*S*(OH)D3.

### 20S(OH)D3 Treatment Modulates Production of Cytokines and Chemokines by Cultured Draining LN Cells

Popliteal and inguinal lymph nodes contain immune cells that traffic to and from the joints in the hind limbs of mice with CIA (50, 60). Cytokine and chemokine production by draining LN cells from mice with CIA, in part, reflect production of cytokines and chemokines by immune cells in arthritic joints (61). To assess whether cytokine and/or chemokine production are changed by treatment of mice with CIA with 20*S*(OH)D3, groups of 9 mice immunized with CII were treated beginning on the day of CII immunization with i.p. administration of 2.4 µg/kg 20*S*(OH)D3 or S.O. daily for 14 days. The mice were then euthanized and isolated popliteal and inguinal LN cells were cultured for 48 h to allow cytokines and chemokines to be released into the culture medium. Levels of Th1, Th2, Th17, and inflammatory cytokines were significantly reduced in the supernatants of the draining LN from mice treated with 20*S*(OH)D3 *vs* S.O. vehicle (**Table 1**). Similar reductions in production of these types of cytokines were observed when we cultured spleen cells from a similarly treated different group of CIA mice (data not shown).

**Table 1:** Treatment of Mice with 20*S*(OH)D3 Modulates Cytokine Production by Lymph node cells.

Treatment of mice	IFNγ	GCSF	GMCSF	IL-17	IL-1α	TNFα
	Pg/ml	Pg/ml	Pg/ml	Pg/ml	Pg/ml	Pg/ml
VEHICLE	6008 ± 174	1859 ± 870	711 ± 277	339 ± 10	33 ± 10	65 ± 13
20*S*(OH)D3	36 ± 20	50 ± 23	23 ± 19	2 ± 1	4 ± 1	3 ≤ 1
p^-^Value	0.001	0.001	0.001	0.001	0.001	0.001

Female DBA/1 Lac J mice (N=6 per group) were treated daily after immunization with CII for 14 days with i.p. 20S(OH)D3 (2.5 µg/kg) in 50 µl sesame oil (S.O.) vehicle or with 50 µl of S.O. vehicle daily. Mice were euthanized and lymph node cells were isolated and cultured as described in Methods for 72 h with 1 µg/ml anti CD3/CD28 monoclonal antibodies for 72 h after which the culture supernatants were harvested and analyzed by multiplex cytokine assay and a Luminex instrument. Differences between groups of vehicle-and 20S(OH)D3-treated mice were analyzed by repeated measures ANOVA.

### Treatment of CIA Mice With 20S(OH)D3 Reduces Percentages of CD4^+^ T Cells and CD19^+^ B Cells From Draining LN Cells and Increases T Regulatory (Treg) CD4^+^T Cell Ratio

Since arthritis in mice with CIA results from a contribution by CD4^+^ T cells and B cells which may be suppressed by CD4^+^CD25^+^Foxp3^+^ Tregs (62), it was important to determine whether percentages of these cell populations were changed by 20*S*(OH)D3 treatment. Groups of DBA/1 mice were immunized with CII and treated with 20*S*(OH)D3 (N=12) or S.O. (N=12) following the same protocol as in the experiment depicted in **Figure 1A**, except mice were euthanized on Day 14 post CII immunization. Draining LN cells were isolated and subjected to analysis by flow cytometry as described under Materials and Methods. The mice treated with 20*S*(OH)D3 had reduced percentages of CD3^+^ CD4^+^ T cells and CD19^+^ B cells (*P*=0.004 and *P*=0.004, respectively) (**Table 2**). We also assessed percentages of CD4^+^CD25+ Foxp3^+^ Tregs in this experiment and found no absolute change in the percentage of these Tregs in the draining LN population (data not shown). However, the ratio of CD4^+^CD25^+^ Foxp3^+^ Tregs to CD3^+^ CD4^+^ T cells in mice with CIA treated with 20*S*(OH)D3 increased, indicating the equilibrium was shifted in favor of less CD3^+^ CD4^+^ T cells compared to this type of Treg (**Table 2**).

**Table 2:** Treatment of CIA mice with 20*S*(OH)D_3_ reduces percentages of CD 4^+^ T cells and B cells in draining lymph nodes while elevating FoxP3 Treg/CD3^+^ CD4^+^ ratio*.

*In Vivo*	Treatment Percentage				
		CD3^+^ CD4^+^ T cells	CD4^+^ CD25^+^ FoxP3 Tregs	CD19^+^ B cells	CD4^+^ CD25 FoxP3^+^ Tregs/CD3^+^ CD4^+^ T cells
Sesame oil (100 µl/day		12.1 ± 0.54	8.8 ± 1.9	17.7 ± 3.5	1.1 ± 0.11
20S(OH)D3 (2.4 µg/kg/day		9.1 ± 0.5	7.0 ± 1.04	11.4 ± 0.7	1.6 ± 0.14
pValue		0.004	NS	0.004	0.023

*Lymph nodes were harvested from DB A/1 LacJ mice immunized with CII and treated daily for 14 days with i.p. S.O. (n=5) or 20S(OH)D3 (2.4 µg/kg) (n=5) dissolved in S.O. Percentage of CD3+ T cells and B cells were determined by flow cytometry after staining with fluorochrome tagged rat anti-mouse CD3, anti-mouse CD19. FoxP3 Tregs were detected by FoxP3 staining kit (eBioscience, San Diego, CA). NS, not significant.

### 20S(OH)D3 Administered by Oral Gavage Suppresses Development of CIA

**Figure 3:**
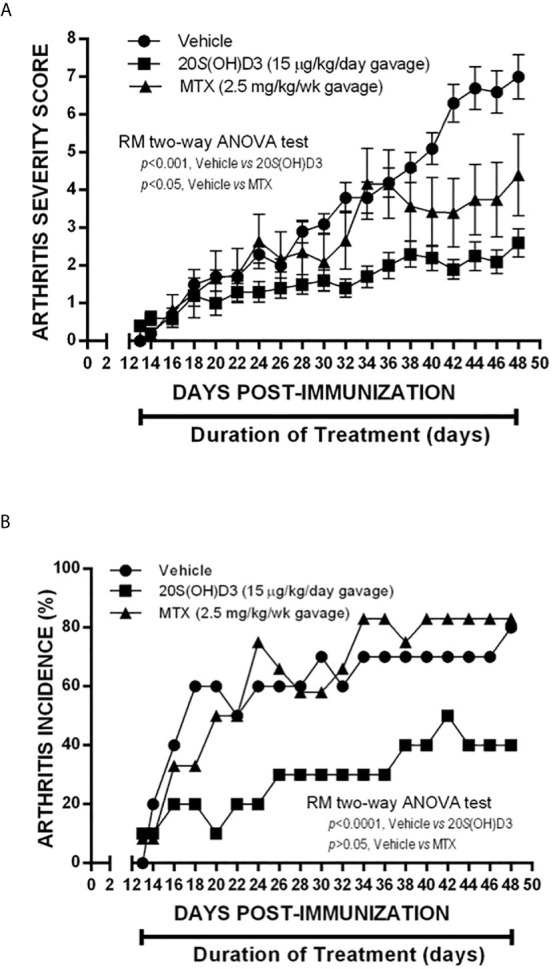
Suppression of CIA by 20*S*(OH)D3 given by gavage: 36 DBA/Lac J female mice 6 weeks of age were immunized with bovine CII (Day 0) and groups of 12 were gavaged orally beginning on day 13 post CII immunization with daily 15 µg/kg 20*S*(OH)D_3 in_ 50 µl 1:5 diluted PG and 50 µl normal saline by oral gavage every 7 days; oral gavage daily with 50 µl 1:5 diluted PG and every 7 days by oral gavage with MTX 2.5 mg/kg dissolved in 50 µl normal saline; or oral gavage daily with 50 µl 1:5 diluted PG and 50 µl normal saline by oral gavage every 7 days. Treatment began at Day 13 after CII immunization and continued through 48 days post CII immunization. Arthritis severity **(A)** and arthritis incidence **(B)** were assessed every 2 days. All mice survived until termination of the experiment.

Since 20*S*(OH)D3 would be given *via* the oral route to humans with RA, if eventually approved to treat this disease, we evaluated whether CIA would be suppressed if 20*S*(OH)D3 were administered *via* the oral route using gavage and how it compared to methotrexate in its ability to suppress CIA. Groups of DBA/1 mice (N=10-12 per group) were immunized with CII and were assigned to different treatments as follows: daily oral gavage 100 µl 1:5 diluted PG containing 15 µg/kg 20*S*(OH)D3 and 100 µl normal saline by oral gavage every 7 days daily oral gavage 100 µl 1:5 diluted PG and 100 µl normal saline by oral gavage every 7 days; and daily oral gavage 100 µl 1:5 diluted PG and every 7 days by oral gavage methotrexate (MTX) 2.5 mg/kg dissolved in 100 µl normal saline. Treatments began at Day 13 Post CII immunization of the mice and continued through 48 days post CII immunization. Arthritis Severity Scores were significantly lower in 20*S*(OH)D3*-* and MTX-treated mice than in PG saline vehicle–treated mice (**Figure 3A**). The incidence of arthritis (percentage of mice with one or more arthritic joints) was also significantly lower in 20*S*(OH)D3–treated mice, but not in MTX-treated mice (**Figure 3B**). This experiment demonstrates that, like MTX (a commonly used medication to treat RA), 20*S*(OH)D3 can suppress CIA when given *via* the oral route.

### 20S(OH)D3 Administered by Gavage Suppresses Established Arthritis in CIA Mice

**Figure 4:**
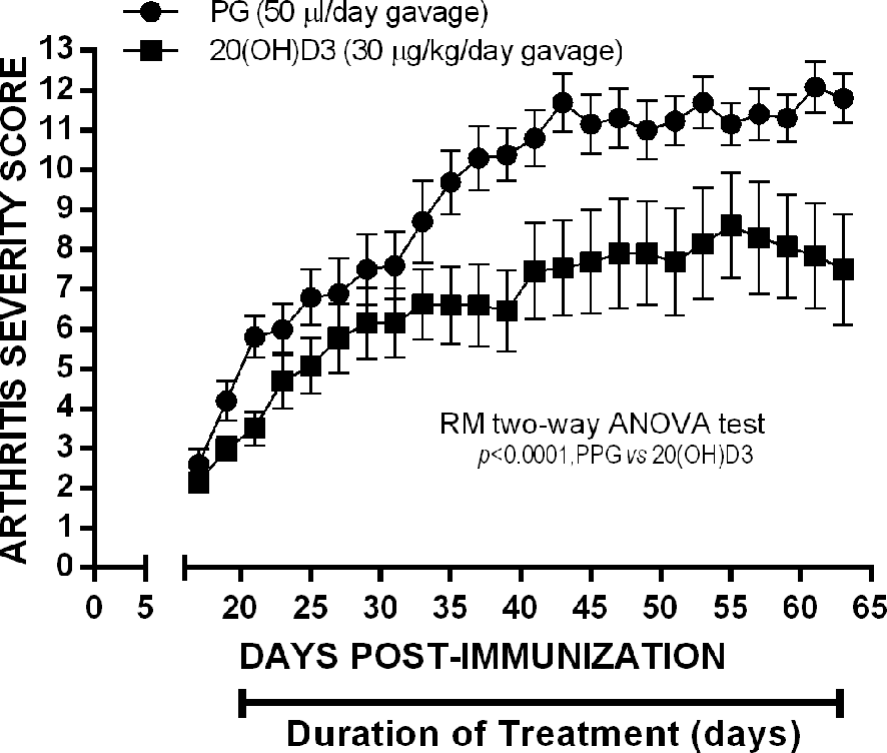
20*S*(OH)D3 given by oral gavage Suppresses established arthritis: 24 6 wk old DBA/1 Lac J female mice were immunized on day 0 with CII and scored for arthritis by a blinded observer every other day for 64 days post CII immunization. Treatment was started day 20 post CII immunization by daily oral gavage with (N=12) 100 µl/day PPG 1:5 dilution in sterile saline (N=12) or 100µl of the PPG 1:5 dilution containing 30 µg/kg 20*S*(OH)D3.

To determine whether 20*S*(OH)D3 would suppress arthritis severity when started later after CII immunization when arthritis is firmly established, we immunized 24 mice with CII and waited until arthritis was present to begin treatment with 20*S*(OH)D3 and S.O. Beginning Day 21, after CII immunization when the mean arthritis severity score was 2.4, we treated 12 of the mice with S.O. administered by daily gavage and 12 with S.O. containing 20*S*(OH)D3 administered by daily gavage at a dose of 30 µg/kg/day. After 46 days treatment, mice were euthanized. As shown in **Figure 4**, mice treated with 20*S*(OH)D3 had less severe arthritis, demonstrating 20*S*(OH)D3 can suppress CIA during the inflammatory phase when anti-CII antibodies play a major role in mediating inflammation in the joints (59, 63). On Day 21 under the conditions we employ, arthritis is highly inflammatory and driven by complement fixing anti-CII antibodies. This suggests 20*S*(OH)D3 also has anti-inflammatory effects.

## Discussion

This is the first demonstration that a natural noncalcemic D3 analog, 20*S*(OH)D3, which is normally produced in humans, suppresses the CIA model of human RA, both clinical arthritis and joint destruction, providing a rationale for further consideration of 20*S*(OH)D3 as a potential mono or adjunctive therapy for RA and other autoimmune diseases. It must be noted that CYP11A1 is also expressed outside the classical steroidogenic organs including skin (64, 65), brain, gastrointestinal tract (66, 67), and immune system (68–71). This suggests that 20*S*(OH)D3 may also be produced by the immune cells. Furthermore, 20*S*(OH)D3 has been detected in honey identifying it as a natural product (42). The mechanisms by which 20*S*(OH)D3 downregulates arthritis severity in the CIA model is likely related to reduction in CD4^+^ T cells, CD19^+^ B cells, anti-CII antibodies, and maintenance of CD4^+^CD24^+^FoxP3^+^Tregs. Treatment with 20(OH) D3 leads to a significant reduction in inflammatory cytokines, likely caused by reduction in the numbers of CD4^+^ T cells together with an increase in the ratio of T reg cells (CD4+CD25+Foxp3+ T cells) to CD3+CD4+ T cells. The decrease in arthritis was also accentuated by a decrease in critical complement-fixing anti-CII antibodies together with a reduction in the number of CD19^+^ B cells. Since pro-inflammatory mediators interact to produce an inflammatory cascade and antibodies against type II collagen lead to destruction of cartilage and bone, these data explain why arthritis is attenuated by 20(OH) D3. These results have some similarities to results obtained using a natural plant product (72). The currently used and/or FDA approved therapeutics to treat RA have the potential to cause mild-to-severe life-threatening adverse events such as bacterial, fungal, or mycobacterium tuberculosis infections, neoplasms such as skin cancers and lymphoma, vasculitis, SLE, MS, and interstitial lung disease, etc (73–75).

20*S*(OH)D3 is produced *in vivo* by the hydroxylation of D3 by CYP11A1 and is non-calcemic in rats and mice (38–41). The serum levels in normal humans of 20*S*(OH)D3 is approximately 5% of *25*(OH)D3. In preclinical studies on C57BL6 mice treated with 20*S*(OH)D3 up to 60 µg/kg given i.p. daily for 3 weeks, there was no evidence of hematologic, renal, or liver toxicity (39). In addition, 20*S*(OH)D3 *in vitro* exhibited anti-inflammatory and pro-differentiatory effects on epidermal cells (32, 34, 76, 77). In contrast, C57B/L6 mice given either 2 µ/kg 1,25(OH)_2_D3 or 25(OH)D3 i.p. daily for 3 weeks displayed hypercalcemia (41). This hypercalcemic property of 1,25(OH)_2_D3 and 25(OH)D3 markedly limits the dosages that can be safely administered to humans on a chronic basis that would be required to treat autoimmune diseases such as RA (78). 20*S*(OH)D_3_ in addition to inhibiting CIA, shares some other biological properties with 1,25(OH)_2_D3 (32). 20S(OH)D3 like 1,25(OH)_2_D_3_ inhibits collagen synthesis by dermal fibroblasts *in vitro*, and at a dose of 3 µ/kg *in vivo* inhibits fibrosis induced by repeated subcutaneous injection of bleomycin into mice (36). 20*S*(OH)D3 also like 1,25(OH)_2_D3 inhibits growth of melanoma cells *in vitro* (37, 40, 79), and it inhibits growth of melanoma at a dose of 30 µg/kg applied daily *in vivo* (37). This is in addition to the aforementioned anti-cancer, pro-differentiation and photoprotective activities of 20*S*(OH)D3 in cells of different origins (80–82). Of significance are anti-inflammatory and immunomodulatory (downregulation of T-cell responses) properties of 20*S*(OH)D3 in conjunction with its ability to decrease NF-kB activity by increasing IkBα levels and inhibiting translocation of NF-kB to the nucleus (76, 81) and inhibit production of IL-17, interferon-γ, TNF-α, and IL-2 (32, 83) and inverse agonism on RORγ (77). These immunomodulatory properties are consistent with the beneficial effect of 20*S*(OH)D3 in the CII-induced arthritis model of RA reported in this paper.

In addition, 20*S*(OH)D3, in contrast to the classical 1,25(OH)_2_D3, is non-calcemic and acts as a biased agonist on the VDR having different interactions with the ligand binding domain in comparison to 1,25(OH)_2_D3 or 1,20(OH)_2_D3 (83, 84). Furthermore, 20*S*(OH)D3 acts as an inverse agonist on RORα and γ (77, 84) and acts as an agonist on the aryl hydrocarbon receptor (AhR) (85). Defining the relative contribution of these nuclear receptors (80, 86) to the reported attenuation of the RA will be addressed in the future studies using transgenic mice with silenced VDR, RORs and AhR receptors.

In summary, we provided for the first time preclinical evidence that 20*S*(OH)D3 can significantly attenuate the progression of arthritis in a murine model of RA *in vivo* through suppression of immune responses by T and B-cells. Thus, we synthesized a novel non-calcemic and nontoxic vitamin D3 hydroxyderivative and demonstrated it to be an excellent candidate for clinical trials in RA and other autoimmune diseases.

## Data Availability Statement

The raw data supporting the conclusions of this article will be made available by the authors, without undue reservation.

## Ethics Statement

The animal study was reviewed and approved by University of Tennessee IACUC Committee.

## Author Contributions

AP and AS conceived and designed the study. RT, TK, WL, SB, LM, and DB contributed data. All authors contributed to the article and approved the submitted version.

## Funding

The work was supported by NIH grants 1RO1 AR052190 and 1R21 AR066505 to AS and AP, 1R01AR073004-01A1, R01AR071189-01A1 and VA merit grant 1I01BX004293-01A1 to AS, and VA Program Project Grant IP1BX001607 to AP and R21 AI149267 to CR and AS.

## Conflict of Interest

The authors declare that the research was conducted in the absence of any commercial or financial relationships that could be construed as a potential conflict of interest.

## In Memoriam

This article is dedicated to our distinguished colleague, the late Professor Arnold E. Postlethwaite, in loving memory of his humanity, humility,integrity and seminal contributions to the broad field of Rheumatology, spanning over five decades.
